# Supplementary material for: Resistance mutation supply modulates the benefit of CRISPR immunity against virulent phages in Pseudomonas aeruginosa
Source: PLoS Biol. 2026 Nov 2;24(11):e3004094. doi: 10.1371/journal.pbio.3004094 (PMC13750652; doi:10.1371/journal.pbio.3004094)
Supplement: Supplementary file 1 [file pbio.3004094.s024.docx]

**EDITORS COMMENTS:**

*You'll see that reviewer #1 is positive, and their requests are all textual and/or presentational. Reviewer #2 is rather more negative, seeing a disconnect between the first and second halves of the manuscript, and regretting the absence of a mechanistic explanation of the benefits of CRISPR. S/he has a list of requests for clarification and more cautious wording. Reviewer #3 is somewhere in the middle, being overall positive, but questioning the statistical treatment, asking why you didn’t measure growth at multiple timepoints, plus a number of requests for clarification.

IMPORTANT: In addition to the reviewers' requests, the Academic Editor also asked me to pass on the following two comments:*

*(i) for clarity, they should mention Pseudomonas at least in their abstract, if not in title*

The abstract and title have been edited to include mention of *Pseudomonas aeruginosa*.

*and (ii) why in Fig. 1 use 2 asterisks (*) to indicate P<0.05? This smells like "statistical inflation".*

We apologise for this oversight. We have now amended the plot to conform to the accepted standards of using one asterisk (*) to refer to p<0.05 in contrast to two asterisks (**) to refer to p<0.01. In no way did we intend to inflate our statistics, indeed we have made effort to be clear and open about our statistical interpretation (e.g., by inclusion of statistical tables in supplementary).

**REVIEWERS' COMMENTS:**

**Reviewer #1:**

*This manuscript is an experimental study of the association of CRISPR effectiveness and the potential of evolving receptor-based resistance. Although such an association seems logical, many factors might affect CRISPR prevalence and experimental studies (like this one) that show particular effects are strongly needed. I have only a few suggestions for the manuscript.* 

**R1.1***. The methods are presented in the end. Thus, it would be good to explain key terms such as efficacy also when first mentioning them in the results.*

We have added explanations of key terms, including efficacy (L78) and escape (L138) when first mentioned in the results section.

**R1.2***. Figure S2 is very informative as it combines receptor classes and efficiency with and without CRISPR. I think that would be suitable as a main figure and could be included in Figure 1. Also, statistical tests between WT and CRISPR KO should be provided for all pairs.*

Figure S2 has been incorporated into Figure 1 (as panel C), including statistical annotation to compare WT and CRISPR KO within the figure and included in full as a statistical table (Table S2).

**R1.3***. l102. "Observed mean CRISPR effects against individual phages were generally predictive of the CRISPR effects against pairs of these phages (Figure 2B)." It is not clear to me how to see this easily from Fig. 2B. Alternatively, maybe a representation similar to Fig. 1B could be shown for CRISPR effect to support this statement. When looking at the extremes, e.g., PA14P2 has high CRISPR effect and PA12P2 has a low effect, but together they have a high effect. In contrast, PA2P1 has high CRISPR effect and PA12P2 has a low effect, but together they have a low effect.*

A figure showing the correlation between CRISPR effects of phage pairs and their average single phage treatments has been added as suggested (Figure 2C). Further, we have added annotation to highlight the phage CRISPR effect signs (+/=) of individual phages in Figure 2B, which we hope helps improve interpretation, and highlights that phages which share the same individual CRISPR effect sign are more likely to maintain that direction of CRISPR effect.

**R1.4.** *In Fig. 2C, it is not completely clear which phages/pairs go into which categories (+, =, ++, ==, +=), maybe that could be marked in Fig. 2B.*

Figure 2B and 2C (now 2D) have been altered to better indicate CRISPR effect categories and groupings, including additional annotation in Figure 2B to show which phages belong to each CRISPR effect category.

**R1.5.** *I found it surprising that spacer acquisition is only mentioned in the Discussion. The results of spacer acquisition and how they were obtained must be mentioned in the Methods and in the Results - even if they are negative.*

Thank you for bringing this oversight to our attention. Spacer acquisition testing has now been added to the methods (L370) and results (L135).

**R1.6.** *Since this is a PLOS Biology "Update Article", the findings of the manuscript should be discussed together with the previous article.*

We have expanded our discussion to highlight the link between our previous article and these new results (L257):

“These results provide greater insight into the mechanisms which drive cross-resistance between phages, whereby asymmetrical cross-resistance between LPS-binding phages [18] may be explained by differences in binding sites across LPS core or O-antigen. Diversity in phage tail structure, even between closely related phages, can thus strongly influence the availability of resistance mutations and the likelihood of broader cross-resistance.“

*Additional comments:*

**R1.7.** *Legend of Fig. 1: mention the statistical test with the p-values. It is unclear what the last line says about the figure.*

Details of the statistical test have been added to the legend of Figure 1. The last line referred to an earlier version of the figure and has been removed – thank you for bringing this to our attention.

**R1.8.** *l142: Give correlation or statistical test for these 2 statements.*

Statistical support for these statements has been added to the text (now L155) and figure legend.

**R1.9.** *First sentence of the discussion - Maybe "identified a novel driver" is strong here, since the findings were consistent with theoretical predictions and the relationship itself is not novel - but showing it experimentally is.* 

This sentence has been edited as follows (L232): “In this study, we provide experimental evidence for an evolutionary modulator of the fitness benefit of CRISPR immunity.”

**R1.10***. Figure S3: Type 2 is slightly mislabeled and should only apply for the last 2 sequences.*

This has now been corrected, thank you.  *Reviewer #2:

This study "Resistance mutation supply modulates the benefit of CRISPR immunity against virulent phages" experimentally tests how the presence of a previously identified CRISPR system affects the ability for PA14 to grow when challenged by a set of 24 environmentally isolated phages. This is certainly an interesting topic, with high mechanistic understanding of CRISPR systems matched by our lack of understanding of the eco-evolutionary significance of these systems. Wright and colleagues use a growth assay - measuring growth in 384-well plates with CRISPR and non-CRISPR strains in the presence of the differing phage strains - to demonstrate against which phage of the panel the CRISPR system is effective. Only against 4 of the 24 phages did the CRISPR system confer a growth advantage to the host. The authors did not determine the basis for resistance in these 4 cases (although they mentioned that PCR was performed in the conclusions). The main message from this first part was that CRISPR could help to preventing phage infection, replication and lysis, although this was relatively rare. 

The second half of the manuscript is associated with the observation that during these growth assays, some replicate cultures showed growth by way of "escape" events (where the host PA14. Generally, these escape events happened against phage in which no advantage of CRISPR was observed. Given the rare nature of CRISPR activity, this observation is not supported by statistics (and this kind of statistics might be difficult and require independence of the CRISPR effect sign and the frequency of escape, when in fact the two values are related). In any case, it was interesting that only against phage where a significant CRISPR effect was observed, was the absence of CRISPR associate with zero escape mutants.*

*The authors then explained this variation in the frequency of escape mutants. They noted a difference in the sequence of the phages' tails and could group the tail sequences based on this sequence identity. They also provided measures of the frequency of resistance mutations to these phages and noted an association between de-novo mutational frequency and the frequency of escape mutant in experimental populations. The authors conclude that different phage strains likely have different targets, and mutational supply varies at these different targets. 

Over all the writing is clear and understandable. There are several points where strong conclusions are extracted without strong support from the data. Generally the experiments seem well performed. The initial OD measure presented in Figure 1 obviously involved a great deal of work and I congratulate the authors on this effort. The presentation of the results until Figure 2B, together with a mechanistic description/test of why CRISPR was beneficial against some strains would be interesting enough for a publication. Unfortunately, the authors could not find this mechanistic cause.

In place, the authors pursued explanation for why there was variation in the frequency of escape mutants against the different phage isolates. What is not clear to this reader is how the first and second part of the manuscript relate to each other - from a mechanistic and/or selective perspective. Yes, the data suggests that CRISPR is beneficial against some of the phage isolates. Yes, the data suggests variation in low rates of resistance mutations is associated with fewer resistant populations (and these resistance populations are less likely when CRISPR could be effective). For me, a clear articulation of the connection between these observations was lacking, but is essential to help understand the message.*

The first and second half of the manuscript are connected because the benefit of CRISPR immunity (quantified in the first half) is negatively associated with the probability of evolving receptor-based resistance by spontaneous mutations (quantified in the second half), which depends on the region of the LPS used as a receptor by the phage. We have added several statements which explain this logic throughout the manuscript (e.g., R2.12, R2.15), that we hope help to better connect the two halves of our results.

**R2.1.** *Furthermore, as detailed further below, there are several "corelations" concluded from the data "a negative correlation between CRISPR effect scores and the frequency of bacterial escape" and "Resistance mutation supply is correlated with differences in tail fibre structure". The authors seem to use correlation in a more general sense (something like associated), but in the absence of a test of correlation (which would be difficult to do), they had better avoid the term entirely.*

These statements have been edited to replace ‘correlated’ with ‘associated’. (e.g., L25, 136)

**R2.2***. Lastly, I'm underwhelmed with the final section of the results. The authors provide a mechanistic reason for the observation in Figs 3A-C by sequencing 5 mutants derived from selection by one of the phage isolates (PA1P3). This doesn't go far to explain difference in mutation frequency between the "groups" (1-3). Aside from sequencing resistant mutants arising from multiple phage isolates (which could be of limited cost if samples were pooled within indexed samples), or the authors could consider using a transposon mutant library (like in ref. 31) to generate diversity and see the genes which (upon loss-of-function) generate resistance to multiple phage isolates.*

To provide stronger mechanistic evidence for different phage receptor targets between Group 1 and Group 2 phages, we have performed an additional coevolution experiment to identify resistance mutations against Group 1 (PELP20 or PA1P3) or Group 2 (14/1 or PA8P2) phages. Twelve replicate populations of PA14ΔCRISPR were propagated with each phage (3 replicates per phage) for 7 days. We sequenced 3 independent resistant clones per phage and consistent with our hypothesis these had gained mutations either in genes encoding the LPS core against Group 1 phage or the O-antigen against Group 2 phage, respectively. See L214, Figures S4&S5 and Table S4.

*Specific points which should be addressed…*

**R2.3.** *Lines 35-36 "suggesting that selection for their gain or loss varies widely in nature" 
This statement would be only true if the presence of CRISPR-systems is variable within given clades. Rephrase to make this clear.*

This sentence (L35) has been edited for clarity, and now reads “CRISPR-Cas systems are encoded by approximately half of sequenced bacterial genomes, yet abundance and system type vary even within clades [2] suggesting that selection for their gain or loss varies widely in nature.”

**R2.4.** *Line 40 "naturally virulent phage" I'm not sure what this means - please explain this term in the text. Is there unnatural virulence? Do the authors mean lytic?*

The phrase ‘naturally virulent’ is used to distinguish virulent phages from temperate phages which have been engineered to become obligately lytic. We feel this is an important distinction as experimental evidence (as referred to below, see comment R2.5), shows that while both formally ‘lytic’, engineered temperate phages and naturally virulent phages have distinctly different relationships with CRISPR-Cas.

**R2.5***. Line 41 "virulent mutants". The term is not clear to me. These are temparate phage that are mutant for lysis? I'm not sure what is intended. Generally, I feel here an expansion of line 40 and line 41 is in order  - this type of variation in immunity in relation to the taget phage is essential for the manuscript and unfortunately I unsure I follow these lines.*

This sentence (L39) has been edited for clarity, and now reads “However, in lab experiments, CRISPR-Cas systems are often ineffective against virulent phage [4–6], in contrast to the highly efficient acquisition of spacers observed against a small number of temperate phages when engineered to become obligately virulent [7–10].”

**R2.6.** *Line 46 "naturally virulent phages" Again, this term doesn't seem specific. Is this referring to lytic phages?* 

Please see response above, R2.4.

**R2.7.** *Lines 87-90 Description of Fig 1A. The relative measure of "efficacy" is not very clear and I think it is explained in methods lines 294-295 "Phage treatment efficacy was calculated as bacterial growth in the presence of each phage treatment relative to phage-free growth" So then this is a relative value based on relative OD at 18hrs growth? I'm therefore for expecting rapacious phage (high infectivity, burst size, small doubling time etc) to cause a "low" value according to the description in lines 294-5. Instead we have a relative measure which seems inverted (the loss of CRISPR increases the presented values). Please restate the exact calculation used to derive this "efficiency" value. Ideally the underlying OD measures - before transformation to a "efficiency" score - are available and plotted in the supplementary file.*

Formulae have been added (L352 for efficacy and L362 for CRISPR effect) to illustrate and differentiate these calculations. Additionally, a brief definition has been added to the results section (L78) and raw OD measurements have been made available in the data deposit (doi: 10.6084/m9.figshare.33071714) and as a supplementary figure (Fig S7).

**R2.8.** *Line 102: "see legend for statistics". In the legend there is reference to the statistics to which is found under the legend for 2B, not 2A. Generally there could be a little more detail on the statistics throughout the manuscript, which is not detailed at all in Fig 1A. Was this a students t-test in 2A? Were the underlying data normal, with similar variance? It seems like there was no Bonferroni correction for the test in 2A?*

More details have been added about statistical testing throughout in figures (e.g., Figure 2A, 3A-C), in the Methods (e.g.,L341, 357) and in the Results section text (e.g., L106, 108). CRISPR effect data were normally distributed. Variance differed among phages but as we used independent tests per phage this is not a concern. Bonferroni correction was not used as the data per phage were independent and not re-sampled.

**R2.9.** *Line 128-132: Comments/questions about these lines and Fig 2C in general. 
        -I'm unsure about the use of the term "negative correlation". The positioning of the five CRISPR effect signs is partially arbitrary (especially in which order =/ == and +/++ are in the plot) and at best the language used here should invoke "associations" and not correlations.*

The term ‘correlation’ has been changed to ‘association (L136) and the connecting lines in Figure 2C (now Figure 2D) have been removed to avoid implying that these constitute a correlation as that was not intended.

**R2.10.**  *-"positive CRISPR effect scores were associated with higher frequencies of escape with versus without CRISPR immunity" I'm either confused about this point OR this is some kind of circular observation. Aren't positive CRISPR effect scores defined by a general ability for the bacteria to grow (likely due to these escape events)? Ie couldn't you define a positive CRISPR score as the higher likelihood of bacterial growth in the presence of a particular phage provided the bacteria has a CRISPR system?*

The text (L139) has been edited to clarify this point (which may also be further helped by editing of Figure 2D, previously 2C), to highlight the intended observation of high rates of escape also in the absence of CRISPR for phages with neutral CRISPR effects, which led us to explore the supply of resistance mutations, and now reads:

“Moreover, both for individual phages and phage pairs, whilst neutral CRISPR effect scores resulted in higher frequencies of escape regardless of CRISPR immunity (Figure 2D; anova: CRISPR effect sign F=13.7, p<0.0001; CRISPR strain F=4.9, p=0.027), positive CRISPR effect scores were only associated with high frequencies of escape in the presence of CRISPR immunity (Fig 2D).”

**R2.11.** *Line 137-138: "Because PA14DCRISPR can only escape phage infection through gain of a surface receptor resistance mutation." What is the reference for this definitive statement? It seems very definitive despite innovation possible given enough mutational supply. Best to temper this by simply deleting "only".*

The sentence has been rephrased to temper the statement (L148), and now reads “In the absence of a functional CRISPR-Cas system, it is presumed that PA14ΔCRISPR relies upon gain of a surface receptor resistance mutation to escape phage infection.”

**R2.12***. Line 139: "supply of such surface receptor resistance mutations decreases". It is not clear what the authors are expecting when they write that the "supply" of surface receptor mutations could alter? These phages seem to target LPS, are they the same LPS structure for every phage? Are they envisaging that the size of the LPS target is variable allowing for variable mutational supply for these resistance mutations? This section could so with a little more expansion and explanation.*

Whilst all 18 phages tested here are known to bind to LPS, they bind to different residues across the O-antigen and exposed core LPS. Therefore, there are a broad range of mutations within the LPS pathway which may alter phage binding in distinct patterns: for example, commonly observed phage-resistance mutations targeting the LPS O-antigen polymerase (Figure S5)*,* may provide resistance against phages which bind the O-antigen but not against phages which bind the LPS core. Therefore, availability of surface receptor resistance mutations may depend on the number of genetic loci in the LPS biosynthesis pathway which modify phage-specific binding sites on the LPS or the viability of mutants in these genes.

The text has been edited to explain this concept more clearly (L149): “Therefore, we hypothesised that CRISPR immunity becomes more beneficial as the supply of such surface receptor resistance mutations decreases, for example, if fewer viable mutational targets in the LPS biosynthesis pathway alter phage-specific binding sites”

**R2.13.** *Line 147: Figure A. Is this data (the proportion of escape events) the same as in 2C? If yes, why are there no data points in Fig 2C equal of greater than 0.6 (like phage 14_1)?*

Thank you for bringing this to our attention. The data originally plotted in Fig 2C was inadvertently calculated from summary data rather than raw data and has been corrected (now Figure 2D).

**R2.14.** *Line 152: Why are only there only 18 sequences here? Are there multiple identical sequences? If yes, is this stated somewhere?*

Only the LPS-binding phages (n=18) are included in this analysis as they have comparable tail fibres. The additional non-LPS binding phages included in earlier analysis (n=8) are excluded here as they do not share genetic similarity in tail fibre proteins, so no comparison is possible.

**R2.15.** *Line 166: "Highly variable". Please specify this is variation between the strains (I was initially assuming this was population-level variation). But more importantly: Explanation/ clarification in the text is required as to why the genetic variation between these phage strains is related to "resistance mutation supply" and the production of de-novo resistance to the phage. I simply don't have an expectation at the beginning of this paragraph why this is the case.  After reading the rest of the results I assume the authors are claiming that variation in the tail proteins suggests different LPS targets between phage "groups" 1 2 and 3 (although there is likely some overlap), and these targets then have their particular rates of resistance mutations. Please share more expectation at the beginning of this paragraph, possibly between the first and second sentence.*

The high variability mentioned indeed indicates variation between our 18 PB1-like phage strains. We have added text to clarify this (L182): “ […] up to ~20% sequence identity across the 18 PB1-like phages (Figure 3D) […]”.

To better set out our rationale, we have added further explanation as suggested (L178): “Variation in phage tail structures, such as tail fibres, is likely to indicate altered LPS-binding activity and influence the identify of phage-specific surface receptor resistance mutations.”

**R2.16.** *Line 175: "functional CRISPR immunity had no effect on bacterial escape" It's not easy to trace this statement back and relate it to the earlier figures. To which phage from group 2 and 3 was there a proven function (ie a benefit shown by a positive CRISPR effect sign)?*

Referencing to the relevant figures (3A-C) has been added to this text, and phage details have been added (L191) to indicate which response we refer to: i.e., that for phage from group 2 (PA8P2, 14/1) and group 3 (PA7P2), escape occurred both in the presence and absence of CRISPR (Figure 3A).

**R2.17.** *Line 199 "variation in resistance mutation supply is mediated by altered hydrogen-bonding" This is confusingly worded and I would avoid "mediated". There's no "mediation" in resistance mutation supply by the phage, which would suggest that the phages are somehow adjusting the production of point mutations in the genome. Rather, the binding targets within the LPS which the different phage bind to are different, and these targets allow variation in mutation frequency, likely resulting from different target sizes and mutation rates. Please rephrase this statement for clarity.*

This sentence has been rephrased as follows (L227): “Taken together, our findings suggest that altered hydrogen-bonding at the C-terminus of the phage tail fibre caused changes in binding targets within the LPS and thus distinct sets of LPS biosynthesis genes wherein mutations provide resistance, resulting in variation in resistance mutation supply. “

**R2.18***. Line 203: I don't really agree with the messaging here. What is the driver? I agree that resistance to phage, caused by CRISPR immunity, is likely beneficial. There seems to be an association (and again, this should probably not be called a correlation) of effective CRISPR activity against phage with a low frequency of escape mutants. I'm not sure the authors can claim this association is "driving a fitness benefit". Rephrase this to more closely reflect the findings presented.*

This sentence has been edited as follows (L232): “In this study, we provide experimental evidence for an evolutionary modulator of the fitness benefit of CRISPR immunity.”

**R2.19.** *Line 232: "No spacer acquisitions could be detected by PCR" This is describing a result which is not mentioned anywhere else, nor how it was done. At a minimum, the amplified region used should be stated, along with the number of replicates with which PCR was performed.*

Thank you for bringing this oversight to our attention. Spacer acquisition testing has now been added to the methods (L370) and results (L135).

**R2.20.** *Line 248-9 "our results suggest increasing phage diversity may inadvertently redirect resistance emergence towards CRISPR immunity". I think the word "redirect" is a metaphor too far. This section is more likely read by a person interested in phage therapy and they would benefit from direct phrasing, such as "our results indicate CRISPR is adaptive for bacteria that do not evolve resistance via mutation. We predict that cocktail therapy may inadvertently select for pathogens expressing such systems" or similar.* 

This sentence has been edited as follows (L281): “A key benefit of phage cocktails is the potential to limit resistance evolution via mutation, however, our results suggest increasing phage diversity may inadvertently select for pathogens encoding CRISPR immunity.”

*Reviewer #3:
[identifies himself as Mark P. Zwart]

Wright et al. report exciting work that sheds light on why the distribution of CRISPR immunity is so patchy across bacterial genomes. Using a panel of virulent phages and Pseudomonas aeruginosa strains with or without a functional CRISPR system, they show that when PA is more likely to evolve receptor-based resistance to a given phage, it is also less likely to benefit from carrying CRISPR immunity. They further investgate why the supply of resistance mutations differs across phages, identifying structural variation in tail proteins as an underlying factor. Overall, the study makes an interesting contribution to a competitive field by moving beyond binary questions of which resistance mechanism will evolve and instead framing resistance as a trade-off governed by mutation supply. Below I provide some comments I hope the authors will find useful in improving the presentation of their work.

Major comments:*

**R3.1.** *The authors approach to include a broad phage panel is a major strength of the paper. Although they describe how the panel was selected in very general terms (i.e., genetic and functional diversity), it would be helpful to provide some more detail here. Was the selection random or opportunistic, where any lytic phage types excluded? Some more detailed explanation and documentation would be helpful to assess how representative this panel is.*

To avoid bias in our selection of phages, we selected all phages in our collection (which in total consists of ~70 lytic phages from varied sources) which were able to either i) cause plaques on PA14 and/or ii) reduce bacterial growth in liquid culture during a 24h growth curve. More details on this methodology has been added to the methods (L310) and a supplementary figure included to show growth curve results for phage screening (Figure S1).

**R3.2.** *Lines 128-130: This is one of the major claims in the paper, the negative correlation between CRISPR effects and bacterial escape from phages. However, the result in only obtained by "inspection", whereas I think the authors need to present a formal statistical test. Furthermore, CRISPR effects are a continuous variable that the authors have decided to bin here, based on significantly positive effects. Why was this choice made, and what happens if one considers both variables as continuous ones?*

As continuous variable both CRISPR effect and frequency of escape are both calculated using phage efficacy data, therefore testing the correlation directly did not seem appropriate. By compromise, we decided to group the phages into CRISPR effect categories. We have added more detail (L141) to the statistical analysis used to interpret this data (now Figure 2D).

**R3.3.** *I see there are benefits to determining growth based on a single timepoint, mainly in terms of simplicity of the analysis. However, so much information on the underlying mechansims is potentially lost (i.e., different underlying trajectories can map to the same value), also informing about the potential causes of growth (i.e., continuous slow growth, discontinuities in growth rates pointing to mutations in bacteria or phage) and underlying host-phage dynamics (i.e., collapses due to phage). It seems like such a lost opportunity to not have gathered the data or used it. I'm sure many readers will feel the same, so can the authors comment on why they chose for this approach?*

This is a limitation imposed by the scales of the experiment. It was simply not feasible to collect additional data points without compromising the sterility of the experiment. This is due to constraints in the experimental setup – the assay design consisted of 50 x 384-well plates which were read through a plate reader using an automated stacker. The plate reader stacker used (Tecan Spark) did not permit use of plate seals, and so each plate in the stack formed the lid for the subsequent plate. Upon repeat readings during a smaller trial experiment, we found that the risk of contamination between wells was high, suggesting that phages aerosolised during plate movements for readings, limiting the option of measuring more than a single time point. Therefore, we decided that the extremely large scale of the experiment (including high levels of replication to capture rare events and a large panel of phage combinations) outweighed the benefit of collecting additional timepoints.

**R3.4.** *Was any type of OD600 vs CFU calibration curve generated for these specific conditions?*

A calibration curve was not used, however the same media was used for the whole experiment and comparisons are controlled within the study.

**R3.5.** *Phage efficacy and CRISPR effect values are calculated as if there is a 1:1 mapping, but I wonder (and doubt) whether that is the case.*

CRISPR effect is calculated from phage efficacy data using the following equation (L362):

$CRISPR effect= \frac{{efficacy}_{WT}}{{efficacy}_{CRISPR KO}}$

*Minor comments:*

**R3.6.** *Csy3 deletion may have more effects than just knocking out CRISPR immunity, and the evolution of resistance in the wildtype strain might therefore reflect other changes (stress responses, a combination of underlying mechanisms including surface receptors?). As the role of the CRISPR system has not been directly verified, I would suggest pointing out that what is generally described as a "CRISPR effect" in the paper might be more complex.*

We have added a comment in the discussion to address this point (L266):

“Further, it is possible that the csy3 mutation (which removes the functionality of CRISPR-Cas immunity here) could have unforeseen additional effects which impact the comparison between bacterial strains.”

**R3.7.** *Could the authors briefly comment on the negative efficacy values in e.g. Fig. 1A? I assume this is simply due to measurement error, or is something else perhaps going on?*

Negative efficacy values indicate higher growth in the presence of phage compared to phage-free conditions. However, we anticipate that this phenomenon is an anomaly relating to growth measurements using OD600 rather than colony forming units: OD600 may be artificially inflated due to changes in cell size or due to production of extracellular polysaccharides in response to phage presence.

**R3.8.** *In Fig. 1 the marking of significance (i.e., ** < p<0.05) is non-standard and inconsistent with notation elsewhere in the paper.*

Statistical notation has been corrected and actual p-values are now given.

**R3.9.** *In Fig. 1 legend, the solid lines referred to in the in the final sentence appear to have been taken out in this version. Delete?*

Thank you for highlighting this oversight, the sentence has now been removed.

**R3.10.** *In the legend of Fig. 2, the significance levels are mentioned under panel B, but they should refer to panel A.*

This has been amended, thank you.

**R3.11.** *I would suggest to break up Fig. 2C into 2 panels, one for the single phage experiments and a second for the pairs of phages. At the authors discretion because maybe this is just the way my mind works, but I prefer to see the separation based on the setup first, and then one based on the outcomes.*

Thank you for this suggestion, we have separated into panels to improve clarity.

**R3.12.** *While the main conclusions of the paper are posited generally, the consideration of resistance mutation supply was only performed for a subset of phages (the eight PB-1 like phages). Therefore, as other escape strategies might exist for other phages, I think this conclusion could be framed a bit more carefully.*

The concluding statement has been edited to clarify that resistance mutation supply is a novel additional factor, on top of previously discussed escape strategies such as defence systems, which may determine efficacy of phage therapy, L285:

“However, our data suggest that understanding the availability of resistance mutations during treatment and how this is influenced by phage genetic and environmental factors will be an important additional factor for predicting how CRISPR immunity will impact the efficacy of phage therapy, enabling development of more robust and efficacious phage-based treatment options.“

**R3.13.** *Key information missing in the methods section (lines 268-294): what was the volume of the cultures used? Given the doubling times and carrying capacity, I assume P. aeruginosa will not start approaching stationary phase within the 18 h time window. But I'm not very familiar with this setup, so is that really the case?*

Further details have now been added to the methods as suggested: the culture volume was 90μl and under these conditions stationary phase would be reached at ~20h (as observed in example growth curves now included as Figure S6).

**R3.14.** *Line 302: "one-sample t-test".*

This has been amended, thank you.

**R3.15.** *Lines 303-305: Please show the distribution (in a supplemental figure?) to show this is a meaningful breakpoint.*

A supplementary figure (S2) showing the efficacy distribution has been added to explain the choice of breakpoint.

**R3.16.** *Availability of the experimental and sequence data: the authors indicate in the PLOS form that they will make all data available, but I would encourage them to then do so during the review stage.*

All experimental data has been made publicly available and can be found on Figshare at doi: 10.6084/m9.figshare.33071714. Sequencing data is available on ENA at study accession PRJEB122815.

**R3.17.** *Supplementary Table 1: great that they included this information. It's probably just me, but I'm not sure what exactly they are doing here. I would find a "model equation" column much more informative than the "Test" column included, because I want to know what exactly they did at each step. Were univariate tests performed first, because some of the variables being added later to the model (i.e., phage identity) have the largest effect on model support. If you are doing forward selection, then it would seem reasonable to include the largest effects first, even if your analysis framework guides you to test by default some predictors anyway?*

An additional column has been added to Supplementary Table 1 giving each model equation and further details have been added to methods (L341) to clarify the statistical methods, which in brief consists of a forward selection stepwise method, carrying over residual variation after each test into the next, building up to the full linear model.
