# Supplementary material for: Resistance mutation supply modulates the benefit of CRISPR immunity against virulent phages in Pseudomonas aeruginosa
Source: PLoS Biol. 2026 Nov 2;24(11):e3004094. doi: 10.1371/journal.pbio.3004094 (PMC13750652; doi:10.1371/journal.pbio.3004094)
Supplement: Supplementary file 2 [file pbio.3004094.s025.docx]

***Editor’s comments:***

*IMPORTANT - please attend to the following:

a) Please address the remaining requests from the reviewers (the Academic Editor, like rev #3, had noticed quite a few typos too).*

Apologies: The manuscript has been given a detailed proofread to catch any remaining typos.  *b) Please supply the numerical values underlying Figs 1ABC, 2ABCD, 3ABCDE, S1, S2, S4, S6, S7, either as a supplementary data file or as a permanent DOI’d deposition.*

### All raw data were already available: via Figshare (doi:10.6084/m9.figshare.33071714) for experimental data; European Nucleotide Archive for sequencing data (project accession: PRJEB122815).

### To further improve data accessibility, we now include all raw data in addition to all numerical values used for plotting (e.g., calculations made based on raw data) as supplementary files.

*c) Please cite the location of the data clearly in all relevant main and supplementary Figure legends, e.g. “The data underlying this Figure can be found in S1 Data” or “The data underlying this Figure can be found; also update your Data Availability Statement accordingly.*

This information has been added.  *d) Please make any custom code available, either as a supplementary file or as part of your data deposition.*

All analysis used published analytical tools which are cited in our methods section.

*e) Please include the URLs of your funders in the Financial Disclosure statement.*

The URL for our funder has been added to our Funding statement.

***Reviewer’s comments:***

***Reviewer #2:*** *It was nice to have a second read over this manuscript, which continues to be an good read. The manuscript deals with several interesting issues (the benefit of crispr, and relative contribution of crispr vs other evolutionary pathways to phage resistance, the influence of different binding targets for the evolution of PA against phage attack etc). Moreover, the MS is a practical contribution to the field and has great relevance for the emerging therapeutic world of phage cocktails.
 
The first round of review has resulted in improvements in the manuscript. There is now clearer guidance for the reader to transition from the initial data on phage efficiency to a mechanistic explanation of observed trends (particularly sections 131-146 and 148-157). The authors had also dropped reference to correlations that were not supported by statistics. The authors now present additional experimental evidence that differences in predicted targets of phage attachment provide an explanation for the frequency of mutational escape. I think they could dive a little deeper with an analysis of the spectrum of mutations found in these further experiments (providing support for their hypotheses (like L149).  However, I won't request more on this point and I'm satisfied with the demonstration of different outer membrane targets mediate escape from these different phage groups. 

I thank the authors for clearly answering my questions and I have limited further questions regarding the points previously raised. I have a few remaining critical questions that have largely resulted from the revision process.

Major remaining questions:

Figure 1A, 1C, 2A, 2D, 3A/B/C - Correct assumptions behind frequentist statistics. The authors have now specified the statistical analysis and used ANOVA (and Post-hoc tests) to compare means in figures 1 and 3 and in figure 2 used independent t-tests "(t-test, population mean significantly higher than 0)". The data underlying these groups in Figures 1A, 1C and Figure 3 do not have a visibly normal distribution. I ran the 1-phage WT data set from Figure 1A with a shapiro-wilk test which showed this distribution was not normal ( W = 0.79729,  p-value < 2.2e-16 ). I'm now very unsure why the authors are using ANOVA tests. The use of these ANOVAs - and not a non-parametric test - requires justification. For both figures 1 and 3, the tests used to test for normality and homoscedasticity should be stated (and ideally in a supplement, the results of these tests would be presented). Similarly, depending on the distribution of the data, the choice of post-hoc tests may well need reconsidering.*

*Regarding statistics in Figure 2A: I had previously requested more statistical details and specifically whether a Bonferroni correction was applied to this data set (see R2.8). The authors have replied to my request with "Variance differed among phages but as we used independent tests per phage this is not a concern. Bonferroni correction was not used as the data per phage were independent and not re-sampled." It is true that one t-test (of whether the mean differs from 0) does not require variance testing. However, the authors are still highlighting instances where - across the 26 single phage treatments - they observe significant differences. This is precisely a situation where a correction for multiple tests is required. If the p-values disappear as a result of that correction, the authors could point to reasonable candidates and repeat the experiments on that limited subset (and of course, not just use the data from these experiments again).  In short: I still maintain the authors require a correction for multiple comparisons in Figure 2A, and the authors should perform this analysis, or provide more specific reasons why they do not require correction for multiple comparisons.*

We apologise for these statistical issues. We have redone the statistical analyses from scratch, as explained in detail below. The revised analyses support our original conclusions in all cases.

**Efficacy analysis (relating to Figure 1)**

The main analysis of efficacy data in Figure 1 uses a linear mixed-effects model (LMM). The LMM fit was assessed using Akaike Information Criterion (Table S1; full model AIC=(-)5593) and overall explained 57.9% of variation in efficacy (R^2^=0.579). The full output from the LMM is included in Supplementary Table 1 for inspection. The raw efficacy data is skewed away from normal (Figure S2). However, LMMs are widely accepted to be highly robust to skewed data (https://besjournals.onlinelibrary.wiley.com/doi/10.1111/2041-210X.13434). Indeed, transforming the data to reduce skew impaired model fit (AIC=550) without altering the significant effects. On balance, we believe that the skew of the raw efficacy data is compensated by the scale of the dataset (N=16,844) and the strong model fit metrics (AIC and R^2^). Given the complexity of the experiment and the robustness of LMM to violations of distributional assumptions, we are confident that the LMM method is the most appropriate analysis of these data.

For Figure 1A/C, the additional ANOVA statistics are no longer necessary in addition to the LMM and have been removed. We now include statistics extracted from the LMM in the main text to support claims relating to these figures (L77-90).

**CRISPR effect analysis (relating to Figure 2)**

On reflection we agree with the reviewer that the 1-sample t-tests were not appropriate and represent multiple testing and apologise for our misunderstanding of the reviewer’s original comment. We have completely redone the statistics for this section.

We have removed the t-tests. In their place we use an LMM to analyse the full CRISPR effect dataset now including both the single-phage and phage-pairs data. The full output of the LMM is provided in new supplementary Table S2 for inspection. The LMM analysis shows significant effects of phage identity and phage interactions explaining variation in CRISPR effect. We believe that this provides a far more powerful and robust analysis than previously.

We have entirely removed the categorical CRISPR effect groupings (that were previously defined using the t-tests) and now analyse and plot CRISPR effect as a continuous variable to explore its correlation with escape frequency (e.g., new Figure 2D). The non-linear relationship between CRISPR effect and escape frequency was modelled as a generalised additive model and the interaction between CRISPR effect and CRISPR strain was interrogated using a chi-squared test. Further, this analysis was performed both with and without the benchmarking phage PA14P2 (new Figure S3) to show that the statistics are robust and not driven solely by known mechanistic interactions with CRISPR.

**Mutation frequency and probability analyses (relating to Figure 3)**

For Figure 3 (A/B/C), we have redone these statistics as follows: Variation in both CRISPR effect and frequency of escape across the subset of 8 LPS-binding phage was confirmed using non-parametric Kruskal-Wallis tests. Then, the relationship between resistance evolution and frequency of escape was measured by linear regression of each resistance metric (mutation frequency and probability of resistance evolution) individually against frequency of escape in the CRISPR KO background.

We hope that the statistical issues identified in the previous version of the manuscript have been satisfactorily resolved. None of the key findings of the paper have changed, but we believe that the new analyses and plots have greater clarity and are more robust. We are grateful to the reviewer for pushing us on this point to improve our analyses. *Minor remaining questions:

Figure 3: There is the label of "a b c" (lower case), which I assume is related to group 1, 2, 3 in the text? If yes, please relabel the figure accordingly (for consistency) or explain why different notations are used in the text and figure.*

This notation has been changed to include asterisks rather than alphabetical labelling of statistically different phage groups.  *Figure 3G/H: It would also be helpful if the region from panel G which panel H is zoomed into (many readers will assume H is a separate structure).*

An outline has now been added to highlight the region in panel G which is shown in panel H.  *Line 182: There a word missing which is not carrying over well from the first clause in the sentence. Perhaps replace with "We identified a highly variable tail protein whose sequence divergence from a chosen representative (PA1P3's gp49), up to ~20% sequence identity divergence across the 18 PB1-like phages (Figure 3D),…". Or, remove the repeated detail entirely and rely more on the first clause "We identified a highly variable tail protein whose sequence divergence from a chosen representative (PA1P3's gp49), up to ~20% across the 18 PB1-like phages (Figure 3D),…"*

This has been rephrased for clarity and now reads: “We identified a highly variable tail protein whose sequence divergence from a chosen representative (PA1P3’s gp49) varied by up to ~20% sequence identity across the 18 PB1-like phages (Figure 3D), which explained differences in escape frequency…” *Line 202: "whereas PA12P2 had 9 fewer or 27 per tail fibre molecule". This needs to be made specific. Is the 9 fewer than PA1P3 or 14/1? I also am clearly misunderstanding something from these lines: "14/1 had 6 fewer donor/acceptor groups than PA1P3 or a total of 18 sites per tail fibre molecule, whereas PA12P2 had 9 fewer or 27 per tail fibre molecule" There some non-transitive arithmetic which I'm simply not understanding. Does the PA1P3 have 36 hydrogen bonds compared to PA12P2 with 27? But then 14/1 should have 30 bonds, (with 6 fewer than PA1P3). I'm confused here and other readers will be too.*

As the protein forms a trimer, the total number of changes per monomer sequence (i.e., 6 or 9 amino acid changes), results in 3x the number of changes in the trimer structure (i.e., 18 or 27). This sentence has been edited for clarity, and now reads: “14/1 had 6 fewer donor/acceptor groups than PA1P3 or a total of 18 fewer sites per trimeric tail fibre molecule, whereas PA12P2 had 9 fewer or 27 fewer per trimeric tail fibre molecule (Figure 3H).” *Lines 227-230 (relating to R2.17): I believe I know what the last sentence of the results in trying to purvey, but the statement is still not very clear. I've read it many times over and got again confused by the wording. I suspect the problem is "the phage tail fibre caused changes in binding targets" Which seems to give some kind of unneeded agency to the tail fibres, whereas they are more simply 'binding different targets'.   Rephrase last sentence of the results.*

This sentence has been edited to read: “Taken together, our findings suggest that altered hydrogen-bonding at the C-terminus of the phage tail fibre resulted in phage binding to different target sites within the LPS and thus distinct sets of LPS biosynthesis genes wherein mutations provide resistance, resulting in variation in resistance mutation supply.”

***Reviewer #3:*** *The authors have reveiwed their submission, clarifying some points in their rebuttal and ammending the manuscript were appropriate.

The manuscript would benefit from careful proofreading, as some errors have crept in (E.g. grammar at lines 47-50, ANOVA [not anova] and Tukey [capitalized] throughout the manuscript).*

Apologies, we have proofread the manuscript again carefully and hopefully caught any further typos.
