# Supplementary material for: Resistance mutation supply modulates the benefit of CRISPR immunity against virulent phages in Pseudomonas aeruginosa
Source: PLoS Biol. 2026 Nov 2;24(11):e3004094. doi: 10.1371/journal.pbio.3004094 (PMC13750652; doi:10.1371/journal.pbio.3004094)
Supplement: Peer review history

# Editor report: Decision Letter 0

**DOI:** 10.1371/journal.pbio.3004094.r001

**Authors:** Roland Roberts

16 Oct 2025

Dear Mike,

Thank you for submitting your manuscript entitled "Resistance mutation supply modulates the benefit of CRISPR immunity against virulent phages" for consideration as a Research Article by PLOS Biology.

Your manuscript has now been evaluated by the PLOS Biology editorial staff, as well as by an academic editor with relevant expertise, and I'm writing to let you know that we would like to send your submission out for external peer review.

IMPORTANT: Please change the article type to "Update Article" when you upload your additional metadata (see next paragraph).

However, before we can send your manuscript to reviewers, we need you to complete your submission by providing the metadata that is required for full assessment. To this end, please login to Editorial Manager where you will find the paper in the 'Submissions Needing Revisions' folder on your homepage. Please click 'Revise Submission' from the Action Links and complete all additional questions in the submission questionnaire.

Once your full submission is complete, your paper will undergo a series of checks in preparation for peer review. After your manuscript has passed the checks it will be sent out for review. To provide the metadata for your submission, please Login to Editorial Manager (https://www.editorialmanager.com/pbiology) within two working days, i.e. by Oct 20 2025 11:59PM.

If your manuscript has been previously peer-reviewed at another journal, PLOS Biology is willing to work with those reviews in order to avoid re-starting the process. Submission of the previous reviews is entirely optional and our ability to use them effectively will depend on the willingness of the previous journal to confirm the content of the reports and share the reviewer identities. Please note that we reserve the right to invite additional reviewers if we consider that additional/independent reviewers are needed, although we aim to avoid this as far as possible. In our experience, working with previous reviews does save time.

If you would like us to consider previous reviewer reports, please edit your cover letter to let us know and include the name of the journal where the work was previously considered and the manuscript ID it was given. In addition, please upload a response to the reviews as a 'Prior Peer Review' file type, which should include the reports in full and a point-by-point reply detailing how you have or plan to address the reviewers' concerns.

During the process of completing your manuscript submission, you will be invited to opt-in to posting your pre-review manuscript as a bioRxiv preprint. Visit http://journals.plos.org/plosbiology/s/preprints for full details. If you consent to posting your current manuscript as a preprint, please upload a single Preprint PDF.

Feel free to email us at plosbiology@plos.org if you have any queries relating to your submission.

Cheers,

Roli

Roland Roberts, PhD

Senior Editor

PLOS Biology

rroberts@plos.org

# Peer review: Decision Letter 1

**DOI:** 10.1371/journal.pbio.3004094.r002

**Authors:** Roland Roberts

5 Dec 2025

Dear Mike,

Thank you for your patience while your manuscript "Resistance mutation supply modulates the benefit of CRISPR immunity against virulent phages" was peer-reviewed at PLOS Biology. It has now been evaluated by the PLOS Biology editors, an Academic Editor with relevant expertise, and by three independent reviewers.

You'll see that reviewer #1 is positive, and their requests are all textual and/or presentational. Reviewer #2 is rather more negative, seeing a disconnect between the first and second halves of the manuscript, and regretting the absence of a mechanistic explanation of the benefits of CRISPR. S/he has a list of requests for clarification and more cautious wording. Reviewer #3 is somewhere in the middle, being overall positive, but questioning the statistical treatment, asking why you didn’t measure growth at multiple timepoints, plus a number of requests for clarification.

IMPORTANT: In addition to the reviewers' requests, the Academic Editor also asked me to pass on the following two comments: (i) for clarity, they should mention Pseudomonas at least in their abstract, if not in title, and (ii) why in Fig. 1 use 2 asterisks (*) to indicate P<0.05? This smells like "statistical inflation".

In light of the reviews, which you will find at the end of this email, we would like to invite you to revise the work to thoroughly address the reviewers' reports.

Given the extent of revision needed, we cannot make a decision about publication until we have seen the revised manuscript and your response to the reviewers' comments. Your revised manuscript is likely to be sent for further evaluation by all or a subset of the reviewers.

In addition to these revisions, you will need to complete some formatting changes, which you will receive in a follow up email. A member of our team will be in touch with a set of requests shortly.

We expect to receive your revised manuscript within 3 months. Please email us (plosbiology@plos.org) if you have any questions or concerns, or would like to request an extension.

At this stage, your manuscript remains formally under active consideration at our journal; please notify us by email if you do not intend to submit a revision so that we may withdraw it.

**IMPORTANT - SUBMITTING YOUR REVISION**

Your revisions should address the specific points made by each reviewer. Please submit the following files along with your revised manuscript:

1. A 'Response to Reviewers' file - this should detail your responses to the editorial requests, present a point-by-point response to all of the reviewers' comments, and indicate the changes made to the manuscript.

*NOTE: In your point-by-point response to the reviewers, please provide the full context of each review. Do not selectively quote paragraphs or sentences to reply to. The entire set of reviewer comments should be present in full and each specific point should be responded to individually, point by point.

You should also cite any additional relevant literature that has been published since the original submission and mention any additional citations in your response.

2. In addition to a clean copy of the manuscript, please also upload a 'track-changes' version of your manuscript that specifies the edits made. This should be uploaded as a "Revised Article with Changes Highlighted" file type.

*Re-submission Checklist*

When you are ready to resubmit your revised manuscript, please refer to this re-submission checklist: https://plos.io/Biology_Checklist

To submit a revised version of your manuscript, please go to https://www.editorialmanager.com/pbiology/ and log in as an Author. Click the link labelled 'Submissions Needing Revision' where you will find your submission record.

Please make sure to read the following important policies and guidelines while preparing your revision:

*Published Peer Review*

Please note while forming your response, if your article is accepted, you may have the opportunity to make the peer review history publicly available. The record will include editor decision letters (with reviews) and your responses to reviewer comments. If eligible, we will contact you to opt in or out. Please see here for more details:

https://blogs.plos.org/plos/2019/05/plos-journals-now-open-for-published-peer-review/

*PLOS Data Policy*

Please note that as a condition of publication PLOS' data policy (http://journals.plos.org/plosbiology/s/data-availability) requires that you make available all data used to draw the conclusions arrived at in your manuscript. If you have not already done so, you must include any data used in your manuscript either in appropriate repositories, within the body of the manuscript, or as supporting information (N.B. this includes any numerical values that were used to generate graphs, histograms etc.). For an example see here: http://www.plosbiology.org/article/info%3Adoi%2F10.1371%2Fjournal.pbio.1001908#s5

*Blot and Gel Data Policy*

We require the original, uncropped and minimally adjusted images supporting all blot and gel results reported in an article's figures or Supporting Information files. We will require these files before a manuscript can be accepted so please prepare them now, if you have not already uploaded them. Please carefully read our guidelines for how to prepare and upload this data: https://journals.plos.org/plosbiology/s/figures#loc-blot-and-gel-reporting-requirements

*Protocols deposition*

To enhance the reproducibility of your results, we recommend that if applicable you deposit your laboratory protocols in protocols.io, where a protocol can be assigned its own identifier (DOI) such that it can be cited independently in the future. Additionally, PLOS ONE offers an option for publishing peer-reviewed Lab Protocol articles, which describe protocols hosted on protocols.io. Read more information on sharing protocols at https://plos.org/protocols?utm_medium=editorial-email&utm_source=authorletters&utm_campaign=protocols

Thank you again for your submission to our journal. We hope that our editorial process has been constructive thus far, and we welcome your feedback at any time. Please don't hesitate to contact us if you have any questions or comments.

Sincerely,

Roli

Roland Roberts, PhD

Senior Editor

PLOS Biology

rroberts@plos.org

------------------------------------

REVIEWERS' COMMENTS:

Reviewer #1:

This manuscript is an experimental study of the association of CRISPR effectiveness and the potential of evolving receptor-based resistance. Although such an association seems logical, many factors might affect CRISPR prevalence and experimental studies (like this one) that show particular effects are strongly needed. I have only a few suggestions for the manuscript.

The methods are presented in the end. Thus, it would be good to explain key terms such as efficacy also when first mentioning them in the results.

Figure S2 is very informative as it combines receptor classes and efficiency with and without CRISPR. I think that would be suitable as a main figure and could be included in Figure 1. Also, statistical tests between WT and CRISPR KO should be provided for all pairs.

l. 102. "Observed mean CRISPR effects against individual phages were generally predictive of the CRISPR effects against pairs of these phages (Figure 2B)." It is not clear to me how to see this easily from Fig. 2B. Alternatively, maybe a representation similar to Fig. 1B could be shown for CRISPR effect to support this statement. When looking at the extremes, e.g., PA14P2 has high CRISPR effect and PA12P2 has a low effect, but together they have a high effect. In contrast, PA2P1 has high CRISPR effect and PA12P2 has a low effect, but together they have a low effect.

In Fig. 2C, it is not completely clear which phages/pairs go into which categories (+, =, ++, ==, +=), maybe that could be marked in Fig. 2B.

I found it surprising that spacer acquisition is only mentioned in the Discussion. The results of spacer acquisition and how they were obtained must be mentioned in the Methods and in the Results - even if they are negative.

Since this is a PLOS Biology "Update Article", the findings of the manuscript should be discussed together with the previous article.

Additional comments:

Legend of Fig. 1: mention the statistical test with the p-values. It is unclear what the last line says about the figure.

l. 142: Give correlation or statistical test for these 2 statements.

First sentence of the discussion - Maybe "identified a novel driver" is strong here, since the findings were consistent with theoretical predictions and the relationship itself is not novel - but showing it experimentally is.

Figure S3: Type 2 is slightly mislabeled and should only apply for the last 2 sequences.

Reviewer #2:

This study "Resistance mutation supply modulates the benefit of CRISPR immunity against virulent phages" experimentally tests how the presence of a previously identified CRISPR system affects the ability for PA14 to grow when challenged by a set of 24 environmentally isolated phages. This is certainly an interesting topic, with high mechanistic understanding of CRISPR systems matched by our lack of understanding of the eco-evolutionary significance of these systems. Wright and colleagues use a growth assay - measuring growth in 384-well plates with CRISPR and non-CRISPR strains in the presence of the differing phage strains - to demonstrate against which phage of the panel the CRISPR system is effective. Only against 4 of the 24 phages did the CRISPR system confer a growth advantage to the host. The authors did not determine the basis for resistance in these 4 cases (although they mentioned that PCR was performed in the conclusions). The main message from this first part was that CRISPR could help to preventing phage infection, replication and lysis, although this was relatively rare.

The second half of the manuscript is associated with the observation that during these growth assays, some replicate cultures showed growth by way of "escape" events (where the host PA14. Generally, these escape events happened against phage in which no advantage of CRISPR was observed. Given the rare nature of CRISPR activity, this observation is not supported by statistics (and this kind of statistics might be difficult and require independence of the CRISPR effect sign and the frequency of escape, when in fact the two values are related). In any case, it was interesting that only against phage where a significant CRISPR effect was observed, was the absence of CRISPR associate with zero escape mutants.

The authors then explained this variation in the frequency of escape mutants. They noted a difference in the sequence of the phages' tails and could group the tail sequences based on this sequence identity. They also provided measures of the frequency of resistance mutations to these phages and noted an association between de-novo mutational frequency and the frequency of escape mutant in experimental populations. The authors conclude that different phage strains likely have different targets, and mutational supply varies at these different targets.

Over all the writing is clear and understandable. There are several points where strong conclusions are extracted without strong support from the data. Generally the experiments seem well performed. The initial OD measure presented in Figure 1 obviously involved a great deal of work and I congratulate the authors on this effort. The presentation of the results until Figure 2B, together with a mechanistic description/test of why CRISPR was beneficial against some strains would be interesting enough for a publication. Unfortunately, the authors could not find this mechanistic cause.

In place, the authors pursued explanation for why there was variation in the frequency of escape mutants against the different phage isolates. What is not clear to this reader is how the first and second part of the manuscript relate to each other - from a mechanistic and/or selective perspective. Yes, the data suggests that CRISPR is beneficial against some of the phage isolates. Yes, the data suggests variation in low rates of resistance mutations is associated with fewer resistant populations (and these resistance populations are less likely when CRISPR could be effective). For me, a clear articulation of the connection between these observations was lacking, but is essential to help understand the message.

Furthermore, as detailed further below, there are several "corelations" concluded from the data "a negative correlation between CRISPR effect scores and the frequency of bacterial escape" and "Resistance mutation supply is correlated with differences in tail fibre structure". The authors seem to use correlation in a more general sense (something like associated), but in the absence of a test of correlation (which would be difficult to do), they had better avoid the term entirely.

Lastly, I'm underwhelmed with the final section of the results. The authors provide a mechanistic reason for the observation in Figs 3A-C by sequencing 5 mutants derived from selection by one of the phage isolates (PA1P3). This doesn't go far to explain difference in mutation frequency between the "groups" (1-3). Aside from sequencing resistant mutants arising from multiple phage isolates (which could be of limited cost if samples were pooled within indexed samples), or the authors could consider using a transposon mutant library (like in ref. 31) to generate diversity and see the genes which (upon loss-of-function) generate resistance to multiple phage isolates.

Specific points which should be addressed…

Lines 35-36 "suggesting that selection for their gain or loss varies widely in nature"

This statement would be only true if the presence of CRISPR-systems is variable within given clades. Rephrase to make this clear.

Line 40 "naturally virulent phage" I'm not sure what this means - please explain this term in the text. Is there unnatural virulence? Do the authors mean lytic?

Line 41 "virulent mutants". The term is not clear to me. These are temparate phage that are mutant for lysis? I'm not sure what is intended. Generally, I feel here an expansion of line 40 and line 41 is in order - this type of variation in immunity in relation to the taget phage is essential for the manuscript and unfortunately I unsure I follow these lines.

Line 46 "naturally virulent phages" Again, this term doesn't seem specific. Is this referring to lytic phages?

Lines 87-90 Description of Fig 1A. The relative measure of "efficacy" is not very clear and I think it is explained in methods lines 294-295 "Phage treatment efficacy was calculated as bacterial growth in the presence of each phage treatment relative to phage-free growth" So then this is a relative value based on relative OD at 18hrs growth? I'm therefore for expecting rapacious phage (high infectivity, burst size, small doubling time etc) to cause a "low" value according to the description in lines 294-5. Instead we have a relative measure which seems inverted (the loss of CRISPR increases the presented values). Please restate the exact calculation used to derive this "efficiency" value. Ideally the underlying OD measures - before transformation to a "efficiency" score - are available and plotted in the supplementary file.

Line 102: "see legend for statistics". In the legend there is reference to the statistics to which is found under the legend for 2B, not 2A. Generally there could be a little more detail on the statistics throughout the manuscript, which is not detailed at all in Fig 1A. Was this a students t-test in 2A? Were the underlying data normal, with similar variance? It seems like there was no Bonferroni correction for the test in 2A?

Line 128-132: Comments/questions about these lines and Fig 2C in general.

-I'm unsure about the use of the term "negative correlation". The positioning of the five CRISPR effect signs is partially arbitrary (especially in which order =/ == and +/++ are in the plot) and at best the language used here should invoke "associations" and not correlations.

-"positive CRISPR effect scores were associated with higher frequencies of escape with versus without CRISPR immunity" I'm either confused about this point OR this is some kind of circular observation. Aren't positive CRISPR effect scores defined by a general ability for the bacteria to grow (likely due to these escape events)? Ie couldn't you define a positive CRISPR score as the higher likelihood of bacterial growth in the presence of a particular phage provided the bacteria has a CRISPR system?

Line 137-138: "Because PA14DCRISPR can only escape phage infection through gain of a surface receptor resistance mutation." What is the reference for this definitive statement? It seems very definitive despite innovation possible given enough mutational supply. Best to temper this by simply deleting "only".

Line 139: "supply of such surface receptor resistance mutations decreases". It is not clear what the authors are expecting when they write that the "supply" of surface receptor mutations could alter? These phages seem to target LPS, are they the same LPS structure for every phage? Are they envisaging that the size of the LPS target is variable allowing for variable mutational supply for these resistance mutations? This section could so with a little more expansion and explanation.

Line 147: Figure A. Is this data (the proportion of escape events) the same as in 2C? If yes, why are there no data points in Fig 2C equal of greater than 0.6 (like phage 14_1)?

Line 152: Why are only there only 18 sequences here? Are there multiple identical sequences? If yes, is this stated somewhere?

Line 166: "Highly variable". Please specify this is variation between the strains (I was initially assuming this was population-level variation). But more importantly: Explanation/ clarification in the text is required as to why the genetic variation between these phage strains is related to "resistance mutation supply" and the production of de-novo resistance to the phage. I simply don't have an expectation at the beginning of this paragraph why this is the case. After reading the rest of the results I assume the authors are claiming that variation in the tail proteins suggests different LPS targets between phage "groups" 1 2 and 3 (although there is likely some overlap), and these targets then have their particular rates of resistance mutations. Please share more expectation at the beginning of this paragraph, possibly between the first and second sentence.

Line 175: "functional CRISPR immunity had no effect on bacterial escape" It's not easy to trace this statement back and relate it to the earlier figures. To which phage from group 2 and 3 was there a proven function (ie a benefit shown by a positive CRISPR effect sign)?

Line 199 "variation in resistance mutation supply is mediated by altered hydrogen-bonding" This is confusingly worded and I would avoid "mediated". There's no "mediation" in resistance mutation supply by the phage, which would suggest that the phages are somehow adjusting the production of point mutations in the genome. Rather, the binding targets within the LPS which the different phage bind to are different, and these targets allow variation in mutation frequency, likely resulting from different target sizes and mutation rates. Please rephrase this statement for clarity.

Line 203: I don't really agree with the messaging here. What is the driver? I agree that resistance to phage, caused by CRISPR immunity, is likely beneficial. There seems to be an association (and again, this should probably not be called a correlation) of effective CRISPR activity against phage with a low frequency of escape mutants. I'm not sure the authors can claim this association is "driving a fitness benefit". Rephrase this to more closely reflect the findings presented.

Line 232: "No spacer acquisitions could be detected by PCR" This is describing a result which is not mentioned anywhere else, nor how it was done. At a minimum, the amplified region used should be stated, along with the number of replicates with which PCR was performed.

Line 248-9 "our results suggest increasing phage diversity may inadvertently redirect resistance emergence towards CRISPR immunity". I think the word "redirect" is a metaphor too far. This section is more likely read by a person interested in phage therapy and they would benefit from direct phrasing, such as "our results indicate CRISPR is adaptive for bacteria that do not evolve resistance via mutation. We predict that cocktail therapy may inadvertently select for pathogens expressing such systems" or similar.

Reviewer #3:

[identifies himself as Mark P. Zwart]

Wright et al. report exciting work that sheds light on why the distribution of CRISPR immunity is so patchy across bacterial genomes. Using a panel of virulent phages and Pseudomonas aeruginosa strains with or without a functional CRISPR system, they show that when PA is more likely to evolve receptor-based resistance to a given phage, it is also less likely to benefit from carrying CRISPR immunity. They further investgate why the supply of resistance mutations differs across phages, identifying structural variation in tail proteins as an underlying factor. Overall, the study makes an interesting contribution to a competitive field by moving beyond binary questions of which resistance mechanism will evolve and instead framing resistance as a trade-off governed by mutation supply. Below I provide some comments I hope the authors will find useful in improving the presentation of their work.

Major comments:

1.The authors approach to include a broad phage panel is a major strength of the paper. Although they describe how the panel was selected in very general terms (i.e., genetic and functional diversity), it would be helpful to provide some more detail here. Was the selection random or opportunistic, where any lytic phage types excluded? Some more detailed explanation and documentation would be helpful to assess how representative this panel is.

2.Lines 128-130: This is one of the major claims in the paper, the negative correlation between CRISPR effects and bacterial escape from phages. However, the result in only obtained by "inspection", whereas I think the authors need to present a formal statistical test. Furthermore, CRISPR effects are a continuous variable that the authors have decided to bin here, based on significantly positive effects. Why was this choice made, and what happens if one considers both variables as continuous ones?

3.I see there are benefits to determining growth based on a single timepoint, mainly in terms of simplicity of the analysis. However, so much information on the underlying mechansims is potentially lost (i.e., different underlying trajectories can map to the same value), also informing about the potential causes of growth (i.e., continuous slow growth, discontinuities in growth rates pointing to mutations in bacteria or phage) and underlying host-phage dynamics (i.e., collapses due to phage). It seems like such a lost opportunity to not have gathered the data or used it. I'm sure many readers will feel the same, so can the authors comment on why they chose for this approach?

4.Was any type of OD600 vs CFU calibration curve generated for these specific conditions? Phage efficacy and CRISPR effect values are calculated as if there is a 1:1 mapping, but I wonder (and doubt) whether that is the case.

Minor comments:

1.Csy3 deletion may have more effects than just knocking out CRISPR immunity, and the evolution of resistance in the wildtype strain might therefore reflect other changes (stress responses, a combination of underlying mechanisms including surface receptors?). As the role of the CRISPR system has not been directly verified, I would suggest pointing out that what is generally described as a "CRISPR effect" in the paper might be more complex.

2.Could the authors briefly comment on the negative efficacy values in e.g. Fig. 1A? I assume this is simply due to measurement error, or is something else perhaps going on?

3.In Fig. 1 the marking of significance (i.e., ** < p<0.05) is non-standard and inconsistent with notation elsewhere in the paper.

4.In Fig. 1 legend, the solid lines referred to in the in the final sentence appear to have been taken out in this version. Delete?

5.In the legend of Fig. 2, the significance levels are mentioned under panel B, but they should refer to panel A.

6.I would suggest to break up Fig. 2C into 2 panels, one for the single phage experiments and a second for the pairs of phages. At the authors discretion because maybe this is just the way my mind works, but I prefer to see the separation based on the setup first, and then one based on the outcomes.

7.While the main conclusions of the paper are posited generally, the consideration of resistance mutation supply was only performed for a subset of phages (the eight PB-1 like phages). Therefore, as other escape strategies might exist for other phages, I think this conclusion could be framed a bit more carefully.

8.Key information missing in the methods section (lines 268-294): what was the volume of the cultures used? Given the doubling times and carrying capacity, I assume P. aeruginosa will not start approaching stationary phase within the 18 h time window. But I'm not very familiar with this setup, so is that really the case?

9.Line 302: "one-sample t-test".

10.Lines 303-305: Please show the distribution (in a supplemental figure?) to show this is a meaningful breakpoint.

11.Availability of the experimental and sequence data: the authors indicate in the PLOS form that they will make all data available, but I would encourage them to then do so during the review stage.

12.Supplementary Table 1: great that they included this information. It's probably just me, but I'm not sure what exactly they are doing here. I would find a "model equation" column much more informative than the "Test" column included, because I want to know what exactly they did at each step. Were univariate tests performed first, because some of the variables being added later to the model (i.e., phage identity) have the largest effect on model support. If you are doing forward selection, then it would seem reasonable to include the largest effects first, even if your analysis framework guides you to test by default some predictors anyway?

# Author comment: Author response to Decision Letter 2

**DOI:** 10.1371/journal.pbio.3004094.r003

30 Jul 2026

Attachment Submitted filename: Response_MB.docx

# Peer review: Decision Letter 2

**DOI:** 10.1371/journal.pbio.3004094.r004

**Authors:** Roland Roberts

8 Sep 2026

Dear Mike,

Thank you for your patience while we considered your revised manuscript "Resistance mutation supply modulates the benefit of CRISPR immunity against virulent phages in Pseudomonas aeruginosa" for consideration as a Update Article at PLOS Biology. Your revised study has now been evaluated by the PLOS Biology editors, the Academic Editor, and two of the original reviewers.

In light of the reviews, which you will find at the end of this email, we are pleased to offer you the opportunity to address the remaining points from the reviewers in a revision that we anticipate should not take you very long. We will then assess your revised manuscript and your response to the reviewers' comments with our Academic Editor aiming to avoid further rounds of peer-review, although we might need to consult with the reviewers, depending on the nature of the revisions.

IMPORTANT - please attend to the following:

a) Please address the remaining requests from the reviewers (the Academic Editor, like rev #3, had noticed quite a few typos too).

b) Please supply the numerical values underlying Figs 1ABC, 2ABCD, 3ABCDE, S1, S2, S4, S6, S7, either as a supplementary data file or as a permanent DOI’d deposition.

c) Please cite the location of the data clearly in all relevant main and supplementary Figure legends, e.g. “The data underlying this Figure can be found in S1 Data” or “The data underlying this Figure can be found in https://zenodo.org/records/XXXXXXXX"; also update your Data Availability Statement accordingly.

d) Please make any custom code available, either as a supplementary file or as part of your data deposition.

e) Please include the URLs of your funders in the Financial Disclosure statement.

In addition to these revisions, you may need to complete some formatting changes, which you will receive in a follow up email. A member of our team will be in touch with a set of requests shortly. If you do not receive a separate email within a few days, please assume that checks have been completed, and no additional changes are required.

We expect to receive your revised manuscript within 1 month. Please email us (plosbiology@plos.org) if you have any questions or concerns, or would like to request an extension.

At this stage, your manuscript remains formally under active consideration at our journal; please notify us by email if you do not intend to submit a revision so that we withdraw the manuscript.

**IMPORTANT - SUBMITTING YOUR REVISION**

Your revisions should address the specific points made by each reviewer. Please submit the following files along with your revised manuscript:

1. A 'Response to Reviewers' file - this should detail your responses to the editorial requests, present a point-by-point response to all of the reviewers' comments, and indicate the changes made to the manuscript.

*NOTE: In your point-by-point response to the reviewers, please provide the full context of each review. Do not selectively quote paragraphs or sentences to reply to. The entire set of reviewer comments should be present in full and each specific point should be responded to individually.

You should also cite any additional relevant literature that has been published since the original submission and mention any additional citations in your response.

2. In addition to a clean copy of the manuscript, please also upload a 'track-changes' version of your manuscript that specifies the edits made. This should be uploaded as a "Revised Article with Changes Highlighted " file type.

*Revision Checklist*

When you are ready to submit your revised manuscript, please refer to this revision checklist: https://plos.io/Biology_Checklist

To submit a revised version of your manuscript, please go to https://www.editorialmanager.com/pbiology/ and log in as an Author. Click the link labelled 'Submissions Needing Revision' where you will find your submission record.

Please make sure to read the following important policies and guidelines while preparing your revision:

*Published Peer Review*

Please note while forming your response, if your article is accepted, you may have the opportunity to make the peer review history publicly available. The record will include editor decision letters (with reviews) and your responses to reviewer comments. If eligible, we will contact you to opt in or out. Please see here for more details:

https://blogs.plos.org/plos/2019/05/plos-journals-now-open-for-published-peer-review/

*PLOS Data Policy*

Please note that as a condition of publication PLOS' data policy (http://journals.plos.org/plosbiology/s/data-availability) requires that you make available all data used to draw the conclusions arrived at in your manuscript. If you have not already done so, you must include any data used in your manuscript either in appropriate repositories, within the body of the manuscript, or as supporting information (N.B. this includes any numerical values that were used to generate graphs, histograms etc.).

*Mandatory Code Policy*

PLOS Biology requires authors to make all author-generated code publicly available without access restriction at the time of publication, unless specific legal or ethical restrictions prohibit public sharing of code, which should be disclosed (https://journals.plos.org/plosbiology/s/code-availability). Shared code needs to be made accessible for free and should be easy to locate and download. We strongly recommend that all code be deposited in a permanent, public repository that issues citable digital object identifiers (DOI) or other persistent identifiers. Where code has been shared in a non-permanent repository, such as Github or Bitbucket, we ask that a snapshot of the code is archived in a permanent, public repository, for example Zenodo, CodeOcean, or the Software Heritage archive.

*Blot and Gel Data Policy*

We require the original, uncropped and minimally adjusted images supporting all blot and gel results reported in an article's figures or Supporting Information files. We will require these files before a manuscript can be accepted so please prepare them now, if you have not already uploaded them. Please carefully read our guidelines for how to prepare and upload this data: https://journals.plos.org/plosbiology/s/figures#loc-blot-and-gel-reporting-requirements

*Protocols deposition*

To enhance the reproducibility of your results, we recommend that if applicable you deposit your laboratory protocols in protocols.io, where a protocol can be assigned its own identifier (DOI) such that it can be cited independently in the future. Additionally, PLOS ONE offers an option for publishing peer-reviewed Lab Protocol articles, which describe protocols hosted on protocols.io. Read more information on sharing protocols at https://plos.org/protocols?utm_medium=editorial-email&utm_source=authorletters&utm_campaign=protocols

Thank you again for your submission to our journal. We hope that our editorial process has been constructive thus far, and we welcome your feedback at any time. Please don't hesitate to contact us if you have any questions or comments.

Sincerely,

Roli

Roland Roberts, PhD

Senior Editor

PLOS Biology

rroberts@plos.org

----------------------------------------------------------------

REVIEWERS' COMMENTS:

Reviewer #2:

It was nice to have a second read over this manuscript, which continues to be an good read. The manuscript deals with several interesting issues (the benefit of crispr, and relative contribution of crispr vs other evolutionary pathways to phage resistance, the influence of different binding targets for the evolution of PA against phage attack etc). Moreover, the MS is a practical contribution to the field and has great relevance for the emerging therapeutic world of phage cocktails.

The first round of review has resulted in improvements in the manuscript. There is now clearer guidance for the reader to transition from the initial data on phage efficiency to a mechanistic explanation of observed trends (particularly sections 131-146 and 148-157). The authors had also dropped reference to correlations that were not supported by statistics. The authors now present additional experimental evidence that differences in predicted targets of phage attachment provide an explanation for the frequency of mutational escape. I think they could dive a little deeper with an analysis of the spectrum of mutations found in these further experiments (providing support for their hypotheses (like L149). However, I won't request more on this point and I'm satisfied with the demonstration of different outer membrane targets mediate escape from these different phage groups.

I thank the authors for clearly answering my questions and I have limited further questions regarding the points previously raised. I have a few remaining critical questions that have largely resulted from the revision process.

Major remaining questions:

Figure 1A, 1C, 2A, 2D, 3A/B/C - Correct assumptions behind frequentist statistics. The authors have now specified the statistical analysis and used ANOVA (and Post-hoc tests) to compare means in figures 1 and 3 and in figure 2 used independent t-tests "(t-test, population mean significantly higher than 0)". The data underlying these groups in Figures 1A, 1C and Figure 3 do not have a visibly normal distribution. I ran the 1-phage WT data set from Figure 1A with a shapiro-wilk test which showed this distribution was not normal ( W = 0.79729, p-value < 2.2e-16 ). I'm now very unsure why the authors are using ANOVA tests. The use of these ANOVAs - and not a non-parametric test - requires justification. For both figures 1 and 3, the tests used to test for normality and homoscedasticity should be stated (and ideally in a supplement, the results of these tests would be presented). Similarly, depending on the distribution of the data, the choice of post-hoc tests may well need reconsidering.

Regarding statistics in Figure 2A: I had previously requested more statistical details and specifically whether a Bonferroni correction was applied to this data set (see R2.8). The authors have replied to my request with "Variance differed among phages but as we used independent tests per phage this is not a concern. Bonferroni correction was not used as the data per phage were independent and not re-sampled." It is true that one t-test (of whether the mean differs from 0) does not require variance testing. However, the authors are still highlighting instances where - across the 26 single phage treatments - they observe significant differences. This is precisely a situation where a correction for multiple tests is required. If the p-values disappear as a result of that correction, the authors could point to reasonable candidates and repeat the experiments on that limited subset (and of course, not just use the data from these experiments again). In short: I still maintain the authors require a correction for multiple comparisons in Figure 2A, and the authors should perform this analysis, or provide more specific reasons why they do not require correction for multiple comparisons.

Minor remaining questions:

Figure 3: There is the label of "a b c" (lower case), which I assume is related to group 1, 2, 3 in the text? If yes, please relabel the figure accordingly (for consistency) or explain why different notations are used in the text and figure.

Figure 3G/H: It would also be helpful if the region from panel G which panel H is zoomed into (many readers will assume H is a separate structure).

Line 182: There a word missing which is not carrying over well from the first clause in the sentence. Perhaps replace with "We identified a highly variable tail protein whose sequence divergence from a chosen representative (PA1P3's gp49), up to ~20% sequence identity divergence across the 18 PB1-like phages (Figure 3D),…". Or, remove the repeated detail entirely and rely more on the first clause "We identified a highly variable tail protein whose sequence divergence from a chosen representative (PA1P3's gp49), up to ~20% across the 18 PB1-like phages (Figure 3D),…"

Line 202: "whereas PA12P2 had 9 fewer or 27 per tail fibre molecule". This needs to be made specific. Is the 9 fewer than PA1P3 or 14/1? I also am clearly misunderstanding something from these lines: "14/1 had 6 fewer donor/acceptor groups than PA1P3 or a total of 18 sites per tail fibre molecule, whereas PA12P2 had 9 fewer or 27 per tail fibre molecule" There some non-transitive arithmetic which I'm simply not understanding. Does the PA1P3 have 36 hydrogen bonds compared to PA12P2 with 27? But then 14/1 should have 30 bonds, (with 6 fewer than PA1P3). I'm confused here and other readers will be too.

Lines 227-230 (relating to R2.17): I believe I know what the last sentence of the results in trying to purvey, but the statement is still not very clear. I've read it many times over and got again confused by the wording. I suspect the problem is "the phage tail fibre caused changes in binding targets" Which seems to give some kind of unneeded agency to the tail fibres, whereas they are more simply 'binding different targets'. Rephrase last sentence of the results.

Reviewer #3:

The authors have reveiwed their submission, clarifying some points in their rebuttal and ammending the manuscript were appropriate.

The manuscript would benefit from careful proofreading, as some errors have crept in (E.g. grammar at lines 47-50, ANOVA [not anova] and Tukey [capitalized] throughout the manuscript).

# Author comment: Author response to Decision Letter 3

**DOI:** 10.1371/journal.pbio.3004094.r005

6 Oct 2026

Attachment Submitted filename: Response.docx

# Editor report: Decision Letter 3

**DOI:** 10.1371/journal.pbio.3004094.r006

**Authors:** Roland Roberts

19 Oct 2026

Dear Mike,

Thank you for the submission of your revised Update Article "Resistance mutation supply modulates the benefit of CRISPR immunity against virulent phages in Pseudomonas aeruginosa" for publication in PLOS Biology. On behalf of my colleagues and the Academic Editor, Arjan de Visser, I'm pleased to say that we can in principle accept your manuscript for publication, provided you address any remaining formatting and reporting issues. These will be detailed in an email you should receive within 2-3 business days from our colleagues in the journal operations team; no action is required from you until then. Please note that we will not be able to formally accept your manuscript and schedule it for publication until you have completed any requested changes.

Please take a minute to log into Editorial Manager at http://www.editorialmanager.com/pbiology/, click the "Update My Information" link at the top of the page, and update your user information to ensure an efficient production process.

PRESS: We frequently collaborate with press offices. If your institution or institutions have a press office, please notify them about your upcoming paper at this point, to enable them to help maximise its impact. If the press office is planning to promote your findings, we would be grateful if they could coordinate with biologypress@plos.org. If you have previously opted in to the early version process, we ask that you notify us immediately of any press plans so that we may opt out on your behalf.

We also ask that you take this opportunity to read our Embargo Policy regarding the discussion, promotion and media coverage of work that is yet to be published by PLOS. As your manuscript is not yet published, it is bound by the conditions of our Embargo Policy. Please be aware that this policy is in place both to ensure that any press coverage of your article is fully substantiated and to provide a direct link between such coverage and the published work. For full details of our Embargo Policy, please visit http://www.plos.org/about/media-inquiries/embargo-policy/.

Thank you again for choosing PLOS Biology for publication and supporting Open Access publishing. We look forward to publishing your study.

Sincerely,

Roli

Roland G Roberts, PhD, PhD

Senior Editor

PLOS Biology

rroberts@plos.org
